# Uridylation of the histone mRNA stem-loop weakens binding interactions with SLBP while maintaining interactions with 3’hExo

**DOI:** 10.1080/15476286.2023.2171760

**Published:** 2023-07-30

**Authors:** Morgan Shine, Sarah E. Harris, Kendy A. Pellegrene, Adam H. Kensinger, Mihaela Rita Mihailescu, Jeffrey D. Evanseck, Patrick E. Lackey

**Affiliations:** a Department of Biochemistry and Chemistry, Westminster College, New Wilmington, PA, USA; b Department of Molecular Biophysics and Biochemistry, Yale University, New Haven, CT, USA; c Department of Biochemistry and Biophysics, University of North Carolina at Chapel Hill, Chapel Hill, NC, USA; d Department of Chemistry and Biochemistry and Center for Computational Sciences, Duquesne University, Pittsburgh, PA, USA

**Keywords:** Histone mRNA, RNA degradation, RNA binding proteins, molecular dynamics

## Abstract

Histone mRNA degradation is controlled by the unique 3’ stem-loop of histone mRNA and the stem-loop binding protein (SLBP). As part of this process, the 3’ stem-loop is trimmed by the histone-specific 3’ exonuclease (3’hExo) and uridylated by the terminal uridylyl transferase 7 (TUT7), creating partially degraded intermediates with short uridylations. The role of these uridylations in degradation is not fully understood. Our work examines changes in the stability of the ternary complex created by trimming and uridylation of the stem-loop to better understand the role of this process in the histone mRNA life cycle. In this study, we used fluorescence polarization and electrophoretic mobility shift assays to demonstrate that both SLBP and 3’hExo can bind to uridylated and partially degraded stem-loop intermediates, although with lower affinity. We further characterized this complex by performing 1-µs molecular dynamics simulations using the AMBER force field and Nanoscale Molecular Dynamics (NAMD). These simulations show that while uridylation helps maintain the overall shape of the stem-loop, the combination of uridylation and dephosphorylation of the TPNK motif in SLBP disrupts key RNA–protein interactions. They also demonstrate that uridylation allows 3’hExo to maintain contact with the stem-loop after partial degradation and plays a role in disrupting key base pairs in partially degraded histone mRNA intermediates. Together, these experiments and simulations suggest that trimming by 3’hExo, uridylation, and SLBP dephosphorylation weakens both RNA–protein interactions and the stem-loop itself. Our results further elucidate the role of uridylation and SLBP dephosphorylation in the early stages of histone mRNA degradation.

## Introduction

**Figure 1.**
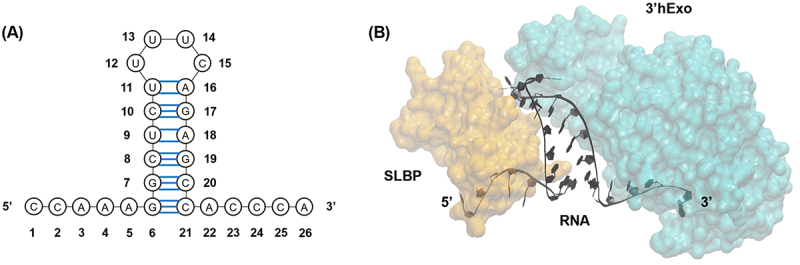
(A) Secondary structure of the highly conserved stem-loop found at histone mRNA 3’ ends. (B) Crystal structure (PDB 4QOZ) [20] of the ternary complex formed by the histone mRNA stem-loop (black), stem-loop binding protein (SLBP) (orange, on the left), and 3’ human exonuclease (3’hExo) (cyan, on the right) .

As the primary protein component of chromatin, histones must be synthesized in carefully regulated amounts to ensure the proper packaging of newly replicated DNA during S phase [1]. Because the most effective way of controlling histone protein synthesis is through histone mRNA levels [2], histone mRNA must be rapidly expressed at the beginning of S phase to meet the high demand for histones and rapidly degraded at the end of S phase to prevent histone overproduction. Dysregulation of this process at any point results in cell fatality [1,3]. These rapid changes in histone mRNA levels are controlled by histone mRNA’s unique 3’ stem-loop. Replication-dependent histone mRNAs are the only known metazoan mRNAs that are not polyadenylated at their 3’ end; instead, they end in a highly conserved stem-loop which is bound by the stem-loop binding protein (SLBP) (Figure 1) [4].

The stem-loop and SLBP control all aspects of histone mRNA metabolism, including processing [4], nuclear export [5], translation [6], and degradation [7]. Histone mRNA is degraded bi-directionally, like other mRNA [8], but because histone mRNA does not have a poly(A) tail, degradation must be initiated by a mechanism other than deadenylation. Histone mRNA is uridylated by the terminal uridylyl transferase 7 (TUT7) in response to the end of DNA synthesis, and thus it has been proposed that uridylation is a part of this degradation mechanism [8,9]. This was proposed in part because the Lsm1-7 ring, a key part of the 5’ to 3’ mRNA degradation pathway [10], can bind to both SLBP and uridylated histone mRNA, provided that the oligo(U) tails are longer than five nucleotides [11]. This previous study only analysed uridylations at the 3’ end of the cytoplasmic histone mRNA, while subsequent high-throughput sequencing of histone mRNA degradation intermediates revealed extensive uridylation of histone mRNA with tails found well into the stem-loop and with many tails much shorter in length than are required for Lsm1-7 binding [9,12].

Previous work suggests that the minimum stem-loop sequence required to bind SLBP with wild-type affinity is a 20-nucleotide RNA molecule with the conserved stem-loop, two flanking adenines on the 5’ side of the stem, and one flanking adenine on the 3’ side [13]. This observation has not been revisited in light of recent high-throughput sequencing experiments [9,12,14] that have characterized both the stem-loop degradation intermediates and short uridylations present during degradation. As such, understanding how both partial degradation and the addition of short oligo(U) tails affect SLBP binding to the stem-loop is one of the goals of this study.

These short oligo(U) tails are tied closely to the activity of the 3’ histone exonuclease (3’hExo, also known as Eri1). This enzyme forms a ternary complex with SLBP and histone mRNA [15] by binding to the 3’ side of the stem-loop [16] after the SLBP-mRNA complex has been exported to the cytoplasm (Figure 1B). Once in this ternary complex, 3’hExo removes two nucleotides from the 3’ end of the stem-loop. If more than two nucleotides are removed from the stem-loop, non-templated uridines are added by TUT7 to restore the 3’ tail to a length of three nucleotides beyond the stem [9,17]. As histone mRNA degradation begins, 3’hExo degrades further into the stem, removing up to seven nucleotides from the 3’ end of the stem-loop [18], while TUT7 continues to add non-templated uridines [9], creating these shorter oligo(U) tails.

**Figure 2.**
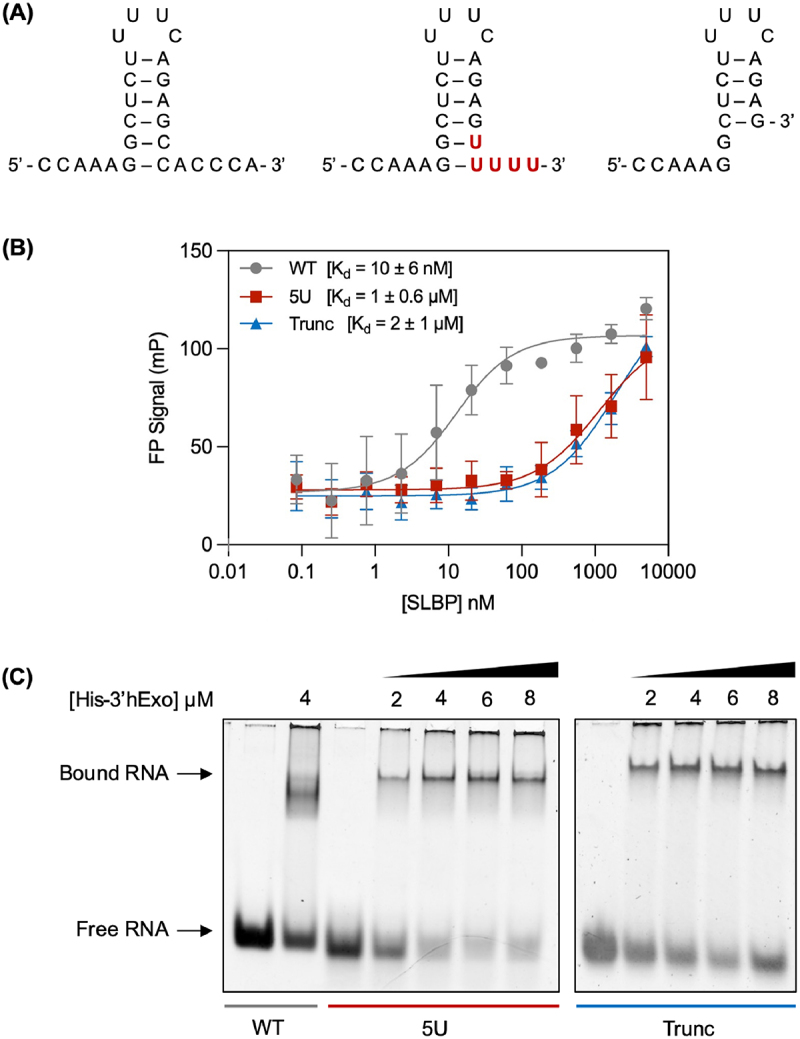
(A) RNA constructs for the wild-type stem-loop (WT) (left), uridylated intermediate (5U) (middle), and truncated stem-loop (Trunc) (right) (B) Fluorescence polarization binding curve for SLBP and each RNA degradation intermediate with relative K_d_ values. (C) 3’hExo binds WT, 5U, and Trunc. Native EMSAs were performed for samples containing 2 µM RNA and increasing concentrations of His-3’hExo in the following RNA to protein molar ratios: 1:0, 1:1, 1:2, 1:3, and 1:4. For the EMSA in **(C)**, BSA (0.010 mg/mL) was added to all samples to prevent non-specific binding interactions. Samples were separated on small 6% polyacrylamide (29:1 acrylamide: bis) gels, which were stained in SYBR Gold.

One of the most common uridylated histone mRNA intermediates shows partial degradation into the stem with seven nucleotides removed from the 3’ end by 3’hExo, but with five uridines added back to the RNA by TUT7 (Figure 2A) [9]. This observation suggests that the intermediate is either important to degradation or that it maintains an interaction with a key regulatory protein like SLBP. Both of these suggestions present logical questions; this uridylated intermediate is too short to bind to the Lsm1-7 ring and was found more often than would be expected after modification by a distributive enzyme [9,11]. SLBP also has specific binding requirements for the stem-loop [13], and it is unclear how the uridylation disrupts the stem-loop structure. By understanding the nature of the interaction, or lack thereof, between these key mRNA intermediates and SLBP, we will gain a better understanding of the first steps of histone mRNA degradation.

Another key element of this ternary complex in the cytoplasm is the phosphorylation of SLBP at Thr171 of the conserved TPNK motif in the protein’s RNA-binding domain, which significantly influences the SLBP-histone mRNA interaction [19]. When phosphorylated at Thr171, SLBP binds the 5’ side of the stem-loop with extremely high affinity and a dissociation constant (K_d_) in the 1–10 nM range [13,19–23]. Dephosphorylation at Thr171 decreases the binding affinity of SLBP for the stem-loop by 7–11-fold [19,23]. Other studies have shown that SLBP is modified by the prolyl isomerase Pin1 and Thr171 is dephosphorylated before histone mRNA degradation begins, potentially tying dephosphorylation to the dissociation of SLBP from the stem-loop at the end of S-phase and facilitating the progression of histone mRNA degradation [24,25].

The goal of this study is to determine how the trimming and uridylation of the histone mRNA stem-loop affects its binding interactions with SLBP and 3’hExo, and how these interactions are modified by the dephosphorylation of SLBP at the TPNK motif. The combination of *in vitro* and *in silico* methods presents a novel approach to investigating these RNA–protein interactions. Experimental biophysical methods, such as fluorescence polarization assays and electrophoretic mobility shift assays (EMSA), can be used to evaluate RNA-protein complex formation, and computational biophysical methods, such as molecular dynamics simulations, can be used to better understand changes in structure and hydrogen bonding interactions.

Molecular dynamics simulations of RNA-protein complexes can be carried out on microsecond time scales [26], which are appropriate for the phenomena being investigated in this study, using high-quality experimental structures as a starting point [27]. For our study, models were constructed using the crystal structures 4QOZ [20] and 4L8R [15] of the cytoplasmic histone ribonucleoprotein complex formed by the human histone mRNA stem-loop, the SLBP RNA-binding domain (RBD), and 3’hExo. The primary difference between these two crystal structures is the phosphorylation state of SLBP: the SLBP used for the 4QOZ crystal structure was produced in baculovirus and is phosphorylated at Thr171 [20], while the SLBP used for the 4L8R structure was produced in *Escherichia coli* and is thus dephosphorylated at Thr171 [15]. While Thr171 phosphorylation has little effect on the overall morphology of the ternary complex, it causes a loop region in SLBP to become ordered and makes SLBP more compact [20]. Simulations with SLBP in both of its phosphorylation states allow for better understanding of these RNA–protein interactions in the context of the histone mRNA degradation mechanism.

Experimentally, fluorescence polarization assays and EMSAs were used to validate the formation of RNA-SLBP and RNA-3’hExo complexes, respectively, with the uridylated intermediate and a truncated stem-loop. Using molecular dynamics simulations, we found that uridylation of the stem-loop allows it to maintain a similar tertiary structure in the ternary complex as the wild-type stem-loop. We also show that uridylation of the stem-loop and dephosphorylation of SLBP weaken RNA-SLBP binding interactions and that uridylation of the stem-loop increases the number of interactions between 3’hExo and the 3’ end of the RNA. Together, these results support a model of histone mRNA degradation initiation in which uridylation of the stem-loop and dephosphorylation of SLBP’s TPNK motif work together to weaken RNA-SLBP interactions and the stem-loop itself while allowing 3’hExo to remain in contact with the 3’ end of the RNA.

## Experimental procedures

### RNA constructs

The wild-type stem-loop and the uridylated intermediate RNA constructs (seen in Figure 2A) were synthesized via T7 RNA polymerase driven *in vitro* transcription reactions [28] using DNA templates obtained from TriLink Biotechnologies. The DNA template for the wild-type stem-loop was designed based on the sequence of the H2A core histone stem-loop, and the DNA template for the uridylated intermediate was designed based on the sequence of one of the most common degradation intermediates identified by Lackey et al. [9]. DNA template sequences are provided in Table S1. RNA samples were purified through denaturing polyacrylamide gel electrophoresis, electrophoretic elution, and dialysis in 10 mM cacodylic acid, pH 6.5. The truncated stem-loop RNA construct (also seen in Figure 2A) was chemically synthesized and purified through desalting by Integrated DNA Technologies.

### Proteins

The Flag-SLBP and 3’hExo were both provided by Dr William Marzluff at the University of North Carolina at Chapel Hill. The proteins were expressed from baculovirus and purified as previously described [16,29,30].

### Fluorescence polarization

Fluorescence polarization assays were performed to measure relative binding affinities of RNA-SLBP complexes with the wild-type stem-loop, the uridylated intermediate, and the truncated stem-loop. 5’ FAM-labelled RNA oligos were ordered from Integrated DNA Technologies (IDT) matching the sequences seen in Figure 2A. Samples were prepared at 5 nM RNA. RNA was boiled for 5 min and snap-cooled for 7 min on dry ice and ethanol to induce the stem-loop conformation. Flag-SLBP was serial-diluted in FP binding buffer (20 mM Tris, pH 8, 50 mM KCl, 0.1 mM EDTA, 10 ng/µL BSA, 50 nM random sequence RNA) to a final concentration of 84.7 pM, 0.254, 0.762, 2.29, 6.86, 20.6, 61.7 nM, 0.187, 0.556, 1.67, or 5 µM. Reactions were incubated at room temperature for 15 min, after which the plates were centrifuged at 1000 x *g* for 1 min. Fluorescence polarization was measured at 25°C using a PHERAstar plate reader (BMG Labtech). Data were fit to a single-site binding model (shown below), and relative K_d_ values were determined.

$$Y_{obs}=Y_{unbound}+\left(Y_{bound}-Y_{unbound}\right)x\frac{{\left[RNA\right]}_{tot}+{\left[SLBP\right]}_{tot}+K_{d}-\sqrt{{\left(-{\left[RNA\right]}_{tot}-{\left[SLBP\right]}_{tot}-K_{d}\right)}^{2}-4{\left[RNA\right]}_{tot}{\left[SLBP\right]}_{tot}}}{2{\left[RNA\right]}_{tot}}$$

### Native electrophoretic mobility shift assays

Native electrophoretic mobility shift assays were carried out to investigate the formation of RNA-SLBP and RNA-3’hExo complexes with the wild-type stem-loop, the uridylated intermediate, and the truncated stem-loop. Samples were prepared with 2 µM RNA and increasing concentrations of Flag-SLBP or His-3’hExo in the following RNA to protein molar ratios: 1:0, 1:1, 1:2, 1:3, and 1:4. RNA was boiled for 5 min and snap-cooled for 7 min on dry ice and ethanol to induce the stem-loop conformation. Bovine serum albumin (BSA) (0.010 mg/mL) was added to all samples to prevent non-specific binding interactions, and samples were prepared in a final volume of 20 µL of ½X TBE (2.5% glycerol, 10 mM KCl, 25 µM EDTA, and 25 mM Tris). RNA and protein were incubated for 15 min at room temperature before being loaded into a small 6% polyacrylamide (29:1 acrylamide: bis) gel. All gels were run at 90 V and 4°C for 45–60 min, stained in SYBR Gold (1X based on 10,000X stock), and visualized with an AlphaImager.

## Model preparation

Molecular dynamics simulations were performed for ternary RNA-protein complexes. Each complex contained either the wild-type stem-loop, the uridylated intermediate, the truncated stem-loop, or an adenylated stem-loop (Figure S1) bound to dephosphorylated SLBP and 3’hExo in the dephosphorylated ternary complex (3**°** complex) or bound to phosphorylated SLBP (pSLBP) and 3’hExo in the phosphorylated ternary complex (P-3**°** complex). RNA-SLBP-3’hExo simulations were initiated with modified versions (described below) of the 2.3-Å-resolution crystal structure of the dephosphorylated ternary complex (PDB 4L8R) [15]. RNA-pSLBP-3’hExo simulations were initiated with modified versions of the 2.6-Å-resolution crystal structure of the phosphorylated ternary complex (PDB 4QOZ) [20]. The 4QOZ and 4L8R structures were first passed through the application PDBFixer [31] to fill in missing residues (residues 117–124 and 271–273 in 3’hExo for 4QOZ; residues 117–123 and 271–273 in 3’hExo and residues 159–164 in SLBP for 4L8R). PDBFixer was also used to convert selenomethionine residues to methionine residues in the 4L8R structure. The uridylated intermediate was modelled by removing nucleotides 25–26 from the wild-type stem-loop PDB coordinates and changing nucleotides 20–24 to uridines. The truncated stem-loop was modelled by removing nucleotides 20–26 from the wild-type stem-loop PDB coordinates. The adenylated stem-loop was modelled by removing nucleotides 25–26 from the wild-type stem-loop PDB coordinates and changing nucleotides 20–24 to adenines. The LEaP module of the AmberTools20 suite was used to prepare the starting topology and coordinates of all simulations [32]. All systems were solvated using the TIP3P water model [33] with a minimal distance of 15 Å from the solute border of the minimal periodic box 80.3 Å × 94.9 Å × 113.3 Å of 22,692 TIP3P waters. Systems were ionized with 20 mM KCl and neutralized with Na^+^ ions to mimic experimental conditions. The AMBER ff99OL3 (ff99 force field with the parmbsc0 α/γ [34] and χOL3 [35] modifications) and ff14SB [36] force fields were applied to describe RNA and protein, respectively. For all systems containing phosphorylated SLBP, the phosaa10 force field [37] was also applied to describe the phosphorylation at Thr171.

## Molecular dynamics simulations

For each system, conjugant gradient minimization (1000 steps), equilibration (10 ns), and production (1 µs) were performed using Nanoscale Molecular Dynamics (NAMD) [38] at the Center for Computational Sciences at Duquesne University. The potential energy and volume of the system were monitored during equilibration and following minimization to ensure that the system was stable using the isothermic-isobaric (NPT) ensemble. During both equilibration and production, a constant temperature of 310 K was maintained using Langevin dynamics [39] with a damping coefficient of 1 ps^−1^. A constant pressure of 1 atm was also maintained using the Langevin piston Nosé-Hoover method [40] with a piston period of 100 fs and a decay time of 50 fs. All bonds involving hydrogen atoms were constrained using the SHAKE algorithm [41], the cut-off distance for nonbonding interactions was defined as 12 Å, and long range electrostatic interactions were calculated using the Particle Mesh Ewald method [42]. Periodic boundary conditions were employed for equilibration and production.

## Trajectory analysis

For each trajectory, the analysis was performed using 1000 frames over the 1 µs of simulation time. RNA and protein structures were visualized using the molecular graphics program Visual Molecular Dynamics (VMD) [43]. Hydrogen bond occupancies were predicted in VMD [43] based on a donor-acceptor distance of 3.5 Å and an angle cut-off of 30°. Root mean square deviation (RMSD) was calculated in VMD [43] for the RNA in each simulation trajectory by aligning all heavy atoms of the nucleotides of the loop and top four base pairs of the stem (nucleotides 8–19) to the stem-loop from the crystal structure of the phosphorylated ternary complex (PDB 4QOZ) [20]. Nucleotides 8–19 were selected for alignment because they exhibit the least variation across all simulations performed in this study, with the average RMSD of nucleotides 8–19 ranging from 3.5 Å to 4.0 Å across all simulations (Figure S2). This baseline deviation of ~3.5 Å compared to the 4QOZ reference RNA may be attributed to the relaxation of the overall structure from its highly restricted conformation in the crystal packing environment. Base pairs in RNA were analysed using Motif Identifier for Nucleic Acids Trajectory (MINT) software [44] and the following criteria: a maximum distance of 3.5 Å between non-hydrogen atoms and a minimum acceptor-hydrogen-donor angle of 150°.

## Results

### SLBP and 3’hExo bind histone mRNA degradation intermediates

Fluorescence polarization assays were performed to evaluate the ability of full-length SLBP to bind to three different RNA constructs (shown in Figure 2A): the wild-type HISTH2AA3 stem-loop sequence (WT), the same sequence that has been truncated through the removal of seven nucleotides by 3’hExo (Trunc), and the uridylated intermediate with five uridines added to the truncated stem-loop (5U). Our results show that SLBP binds the wild-type stem-loop with a K_d_ of about 10 nM, while the truncated and uridylated RNA constructs both exhibit approximately a 100-fold reduction in binding to SLBP when compared to the wild type (Figure 2B). The SLBP used in these experiments was expressed in baculovirus; previous work indicates that baculovirus-expressed SLBP is stoichiometrically phosphorylated at Thr171 [19]. Strikingly, the addition of the uridylated tail did not significantly increase binding of SLBP to the RNA, even though it returns the stem-loop to the previously established ‘minimum’ length for binding [13] and provides an opportunity for G-U base-pairing down the bottom of the stem.

The ability of 3’hExo to bind the degradation intermediates of interest was evaluated through native electrophoretic mobility shift assays (EMSAs). Our results show that 3’hExo is able to bind all three RNA constructs, indicating that trimming and uridylation of the stem-loop does not eliminate 3’hExo binding (Figure 2C). The shift in band intensity from free RNA to bound RNA is more distinct for the uridylated intermediate than the truncated stem-loop, as signified by lighter free RNA bands at high 3’hExo concentrations for the uridylated intermediate. These results qualitatively suggest that 3’hExo binding is more favourable for the uridylated intermediate than the truncated stem-loop.

### The stem of the histone mRNA stem-loop is unchanged by uridylation when bound by SLBP and 3’hExo

To gain a better understanding of the differences in the RNA–protein interactions in the histone mRNA stem-loop-SLBP-3’hExo ternary complex, eight RNA-protein complexes were studied computationally using molecular dynamics simulations. These complexes were modelled based on crystal structures that include the stem-loop, the RNA-binding domain of SLBP, and the SAP and nuclease domains of 3’hExo (a representative image of these complexes is shown in Figure 1B). Of the eight complexes studied, six are based on observed histone mRNA degradation intermediates [9]: the wild-type stem-loop with the SLBP phosphorylated at the TPNK motif and 3’hExo (WT P-3°), the wild-type stem-loop with SLBP dephosphorylated at the TPNK motif and 3’hExo (WT-3°), the uridylated stem-loop with phosphorylated SLBP and 3’hExo (5U P-3°), the uridylated stem-loop with dephosphorylated SLBP and 3’hExo (5U 3°), the truncated stem-loop with phosphorylated SLBP and 3’hExo (Trunc P-3°), and the truncated stem-loop with dephosphorylated SLBP and 3’hExo (Trunc 3°). We also created two complexes with an adenosine tail in place of a uridine tail to better understand the role of the uridines in degradation (Figure S1); these complexes are the adenylated histone mRNA stem-loop with phosphorylated SLBP and 3’hExo (5A P-3°) and the adenylated histone mRNA stem-loop with dephosphorylated SLBP and 3’hExo (5A 3°).

**Figure 3.**
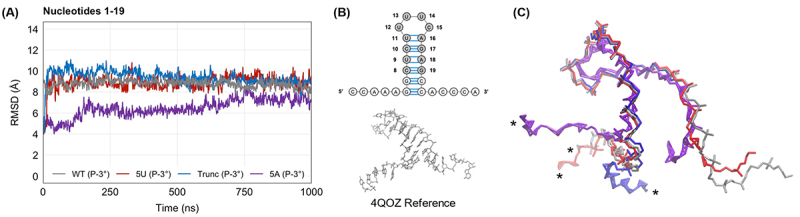
(A) Root mean square deviation (RMSD) for nucleotides 1–19 of the wild-type stem-loop (WT) (grey), the uridylated intermediate (5U) (red), the truncated stem-loop (Trunc) (blue), and the adenylated stem-loop (5A) (purple) in RNA-pSLBP-3’hExo (P-3°) simulations. All heavy atoms of nucleotides 8–19 were aligned to the 4QOZ reference structure, and RMSD was calculated in VMD for all heavy atoms of nucleotides 1–19 based on 1000 frames over the 1000 ns of simulation time. (B) Schematic of the histone mRNA stem-loop with nucleotides used for alignment highlighted in grey (top) and the 4QOZ reference structure used for alignment (bottom). (C) Overlay of average structures of WT (grey), 5U (red), Trunc (blue), and 5A (purple).

Root mean square deviation (RMSD) analysis of the RNA from the four phosphorylated ternary complex simulations was used to study changes in the stability of the stem-loop in the RNA-protein complex after partial degradation and uridylation. Because RMSD works best with complexes of similar size, we used the average RMSD for nucleotides 1–19 of the stem-loop, as these nucleotides are shared by all three stem-loops. Our analysis shows that the average RMSDs of the uridylated intermediate and truncated stem-loop are only 0.1 Å and 0.6 Å higher, respectively, than the wild-type stem-loop, while the average RMSD of the adenylated stem-loop is 2.3 Å lower (Figure 3A). Further analysis revealed that bases 8–19, the base pairs of the stem and the loop itself shared between all three RNA structures, were nearly identical (Figure S2), indicating that the main difference between these three constructs is in the 5’ flanking sequence that precedes the stem.

These differences are also highlighted by the superposition of average structures shown in Figure 3C. Similar results were observed for the dephosphorylated ternary complex simulations (Figure S3). We did not observe any real differences or obvious patterns in weak interactions between SLBP and this 5’ flanking sequence in our hydrogen bond analysis (Table S2). Due to the difference between the constructs used for simulations and the biological histone mRNA, which would extend far beyond these bases in the 5’ direction, these differences in the 5’ flanking sequence are likely not biologically relevant. Notably, our average structure overlay in Figure 3C shows a distinct variation on the 3’ side of the RNA molecule for the adenylated stem-loop relative to the other constructs. As RMSD is not the most appropriate metric to explain this variation due to the major differences in sequence and length of the four stem-loops in this region, we investigate this variation later using different analyses. Overall, our results suggest that the stem of the histone mRNA stem-loop remains unchanged despite trimming and/or uridylation at the 3’ end, while some variation exists in the 5’ and 3’ flanking regions.

### Dephosphorylation of SLBP and uridylation of the stem-loop weaken RNA-SLBP interactions

To look at the effects of uridylation and SLBP dephosphorylation on the stability of this complex, we focused on each protein and their interactions with the histone mRNA stem-loop individually. First, we looked closer at the effect of uridylation and partial degradation of the stem-loop on the interaction between both the phosphorylated and dephosphorylated forms of SLBP. Previous studies [13,45] have shown that SLBP binding requires a guanine at position 7 of the histone mRNA stem-loop. Analysis of the dephosphorylated ternary complex crystal structure by Tan et al. [15] further shows that two hydrogen bonds between the nucleobase of G7 and the guanidinium side chain of Arg181 are the only base-specific hydrogen bonds between the histone mRNA stem-loop and SLBP: G7(N7)-Arg181(NE) and G7(O6)-Arg181(NH2). These observations of the crystal structure confirmed previous observations that Arg181 is necessary for the interaction between SLBP and the stem-loop [46,47].

We calculated occupancy values for all the intermolecular hydrogen bonds in the phosphorylated ternary complex and dephosphorylated ternary complex simulations (Tables S2-S3). In our simulations of the phosphorylated ternary complex, the wild-type and uridylated stem-loops exhibit relatively high occupancy values for the two base-specific G7-Arg181 hydrogen bonds, with values ranging from 31% to 50% for the G7(N7)-Arg181(NE) hydrogen bond and ranging from 50% to 58% for the G7(O6)-Arg181(NH2) hydrogen bond (Table 1). These values are much higher than the values in the truncated and adenylated stem-loops; the range of the two interactions in those constructs is 14–18% and 19–36%, respectively. The low occupancy value for the truncated stem-loop is expected given the experimental results in Figure 2 and the previous research done on SLBP’s binding requirements, but the difference between the uridylated stem-loop, the truncated stem-loop, and the adenylated stem-loop is noteworthy for a number of reasons. One is the biological necessity that the histone mRNA stem-loop must become truncated by 3’hExo before it can become uridylated; thus, it follows that U-tails of this length may function to ‘re-stabilize’ the complex when SLBP is still phosphorylated at the TPNK motif. The observation that this result is not replicated in the adenylated stem-loop suggests a role for the non-canonical G-U base pairs at the bottom of the stem.

**Table 1.** Occupancy values for hydrogen bonds between Arg181 of SLBP and G7 of the stem-loop in RNA-pSLBP-3’hExo (P-3**°**) and RNA-SLBP-3’hExo (3**°**) simulations with the wild-type stem-loop (WT), the uridylated intermediate (5U), the truncated stem-loop (Trunc) and the adenylated stem-loop (5A). The change in occupancy due to SLBP dephosphorylation is shown in the (Δ) column for each stem-loop. Hydrogen bonds were predicted in VMD based on a donor-acceptor distance of 3.5 Å and an angle cut-off of 30°.

Nucleotide	Amino acid	WT (P-3°)	WT (3°)	WT (Δ)	5U (P-3°)	5U (3°)	5U (Δ)	Trunc (P-3°)	Trunc (3°)	Trunc (Δ)	5A (P-3°)	5A (3°)	5A (Δ)
G7 (N7)	Arg181 (NE)	31.3%	29.8%	−1.50%	50.1%	23.2%	−26.9%	18.0%	11.0%	−7.00%	14.2%	46.6%	32.4%
G7 (O6)	Arg181 (NH2)	57.8%	76.2%	18.4%	49.5%	28.2%	−21.3%	19.4%	12.5%	−6.90%	35.7%	69.9%	34.2%

The dephosphorylation of SLBP has a negative effect on these G7-Arg181 interactions in the uridylated stem-loop, as the G7(N7)-Arg181(NE) bond occupancy drops from 50% to 23%, and the G7(O6)-Arg181(NH2) bond drops from 49.5% to 28.2%. This effect is not seen in the wild-type stem-loop and the occupancy value increases with the oligo(A) tail after dephosphorylation, which indicates that the decrease seen in the uridylated stem-loop is an effect specific to the U-tail itself and cannot be replicated by any non-templated nucleotide addition.

### Uridylation increases the flexibility of the base of the stem

Given the importance of the G7 nucleotide and its interactions with SLBP for RNA-protein binding, we further investigated the effect of uridylation of the stem-loop by examining how the oligo(U) tail affects the stability of the stem-loop itself while in the ternary complex. This is especially important as the C20 and C21 bases are replaced with uridines, creating two G-U base pairs at the bottom of the stem. As the G-U base pair has a slightly different geometry than a canonical Watson–Crick base pair [48], replacing G-C pairs with G-U pairs may alter the conformation of the stem-loop itself and explain the changes in contact between Arg181 and G7 in the uridylated intermediate discussed above. To evaluate the stability of these base pairs in our simulations, we utilized the MINT software package [44], which identifies base pairs and calculates the percentage of frames throughout a trajectory for which they are observed. Our MINT analysis shows that a G7-U20 base pair forms in the 5U-pSLBP-3’hExo simulation with a frequency of 47.3%; however, the frequency of this base pair drops to 20.0% when SLBP is dephosphorylated (Table 2). The uridylated intermediate also exhibits a G6-U21 base pair in the 5U-pSLBP-3’hExo simulation, appearing in 67.2% of the simulation (Table 2). This G6-U21 base pair is slightly reduced to 61.5% in the 5U-SLBP-3’hExo simulation (Table 2). These values, while reduced from the 98–99% occupancy values exhibited by the canonical base-pairing in the wild-type stem-loop, are also much higher than the minimal occupancies shown by the adenylated version, again helping to illustrate the utility of an oligo(U) tail of this length added in this position. This base pair analysis further suggests that, as demonstrated in Figure 3, although the overall shape of the stem-loop is similar in the wild-type and uridylated stem-loops, the bottom two base pairs in the uridylated intermediate are weaker than in the wild-type stem-loop, allowing G6 and G7 to have greater flexibility that may result in the change in interaction at the G7 base pair when SLBP is dephosphorylated.

**Table 2.** Percentages of frames for which base pairs are observed in the phosphorylated ternary complex (P-3°) and dephosphorylated ternary complex (3°) simulations with the wild-type stem-loop (WT), the uridylated intermediate (5U), the truncated stem-loop (Trunc), and the adenylated stem-loop (5A). Base pair analysis was conducted with MINT software [44]. Outliers (shaded in grey) were identified using a box plot (Figure S4) and confirmed using a Grubbs’ outlier test (Table S4).

Base pair	WT (P-3°)	WT (3°)	5U (P-3°)	5U (3°)	Trunc (P-3°)	Trunc (3°)	5A (P-3°)	5A (3°)	Average	Average (without outlier)
U11 – A16	86.2%	86.4%	84.1%	85.7%	86.1%	82.7%	83.4%	82.5%	85 ± 2%	NA
C10 – G17	98.8%	99.0%	99.2%	99.0%	99.3%	98.8%	98.2%	99.2%	98.9 ± 0.4%	NA
U9 – A18	92.1%	92.8%	89.4%	89.4%	83.9%	90.3%	91.9%	91.8%	90. ± 3%	NA
C8 – G19	98.1%	98.3%	98.0%	61.6%	97.6%	90.3%	93.5%	94.2%	91 ± 12%	96 ± 3%
G7 – C/U/A20	98.8%	99.1%	47.3%	20.0%	NA	NA	2.20%	4.30%	45 ± 45%	NA
G6 – C/U/A21	99.0%	98.1%	67.2%	61.5%	NA	NA	0.10%	11.0%	56 ± 42%	NA

**Figure 4.**
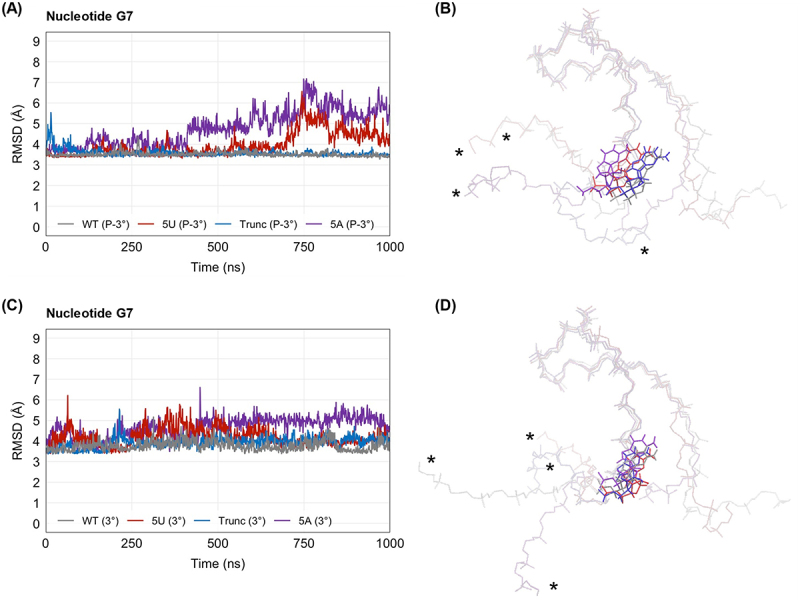
Root mean square deviation (RMSD) of nucleotide G7 in the wild-type stem-loop (WT), the uridylated intermediate (5U), the truncated stem-loop (Trunc), and the adenylated stem-loop (5A) in (A) phosphorylated ternary complex simulations (P-3°) and in (C) dephosphorylated ternary complex simulations (3°). Overlays of the representative RNA structures from each (B) phosphorylated ternary complex simulation and each (D) dephosphorylation ternary complex simulation are shown. The wild-type stem-loop is shown in grey, the uridylated intermediate is shown in red, the truncated stem-loop is shown in blue, and the adenylated stem-loop is shown in purple. G7 and the nucleic backbone are depicted in liquorice model. The 5’ ends of the RNA are on the left side of the molecule and labelled with an asterisk (*). The 3’ ends are on the right side.

To further investigate this, we performed RMSD analysis for nucleotide G7 (Figure 4), which shows that G7 in the RNA constructs adopts three major conformations across the simulations of the phosphorylated ternary complex and two major conformations across the simulations of the dephosphorylated ternary complex. In the phosphorylated ternary complex simulations, the wild-type and truncated stem-loops adopt almost identical conformations of G7, with average RMSDs of 3.5 ± 0.1 Å and 3.6 ± 0.2 Å (Figure 4A). In contrast, the uridylated intermediate and adenylated stem-loop adopt separate G7 conformations from the other RNA molecules, differing by 0.5 Å and 1.3 Å in average RMSD from the wild-type stem-loop, respectively (Figure 4A). More specifically, the uridylated intermediate diverges from the wild-type stem-loop near 700 ns, resulting in a maximum difference of 3.1 Å at 745 ns (Figure 4A). Similarly, the adenylated stem-loop diverges from the wild-type near 450 ns, with a maximum difference of 3.7 Å at 758 ns (Figure 4A). As Table 1 shows, the wild-type and uridylated stem-loops both maintain high hydrogen bond contacts between G7 and Arg181 in the phosphorylated ternary complex, but the specific occupancies of the two bonds between the RNA and protein are not identical. This conformational shift in the G7 base may arise from the aforementioned geometry shift of the G-U base pair, coupled with SLBP maintaining contact at the base through Arg181.

In the dephosphorylated ternary complex simulations, the wild-type, uridylated, and truncated stem-loops adopt one major conformation at G7 by the end of the 1000 ns, with average RMSDs of 3.8 ± 0.3 Å, 4.2 ± 0.4 Å, and 3.9 ± 0.3 Å, respectively, while G7 of the adenylated stem-loop adopts a separate conformation, differing by 0.9 Å in average RMSD from the wild-type stem-loop (Figure 4C). In both cases, G7 of the adenylated stem-loop differs most from the other RNA constructs, and the wild-type and truncated stem-loops exhibit the most similar G7 positions. Dephosphorylation of SLBP appears to have the largest effect on G7 position in the uridylated intermediate by converting it from a distinct conformation in the phosphorylated ternary complex to a similar conformation compared to the wild-type stem-loop in the dephosphorylated ternary complex. This shift in G7 conformation in the uridylated intermediate after SLBP is dephosphorylated may help explain the large change in hydrogen bond occupancy between the protein and the uridylated stem-loop caused by dephosphorylation of SLBP, as seen in Table 1. As shown by the truncated stem-loop in Table 1 and Figure 2A, a low RMSD at G7 does not necessarily correlate with a high hydrogen bond occupancy between the nucleotide and Arg181.

### Stem-loop uridylation affects histone mRNA’s ability to interact with 3’hExo

**Figure 5.**
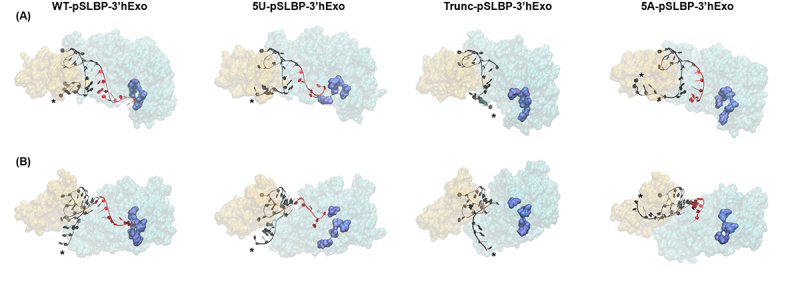
(A) Representative frames of the stem-loop (black), SLBP (orange), and 3’hExo (cyan) from the phosphorylated ternary complex simulations with the wild-type stem-loop (WT) (column 1), the uridylated intermediate (5U) (column 2), the truncated stem-loop (Trunc) (column 3), and the adenylated stem-loop (5A) (column 4). The catalytic residues from the active site of 3’hExo (residues Asp134, Glu136, Asp234, His293, and Asp298) are shown in dark blue, and the 3’ ends of the RNA (nucleotides 20–26) are shown in red. (B) Rotated view of the representative frames highlighting the orientation of the 5’ flanking sequence (*) of the stem-loop.

To better understand the effects we see on the stem-loop discussed above, we also examined the effect that uridylation of histone mRNA has on the interactions between the RNA and 3’hExo. As shown in Figure 5, representative frames from the simulations involving the phosphorylated complex show that the 3’ end of the wild-type stem-loop is positioned in close proximity to the amino acids previously identified as being required for 3’hExo’s catalytic activity: Asp134, Glu136, Asp234, His293, and Asp298 [49]. The uridylated intermediate also shows a similar effect, with the U-tail pulled in the direction of the catalytic site in a way that the A-tail of the adenylated stem-loop is not. This aligns with previous observations [18] that 3’hExo can degrade and may even prefer uridylated intermediates as substrates for degradation.

To quantify this effect, we counted the weak interactions between 3’hExo and the 3’ end of the stem-loop, looking for individual interactions between nucleotides and the amino acid residues, as well as the total number of simulation frames that each interaction appeared in. The total interactions are summarized in Table 3, while the individual interactions are fully reported in Table S3. These data confirm the observations made above about Figure 5 and help to explain the exact interactions between RNA and protein at the different 3’ ends.

**Table 3.** The total number of frames containing interactions between 3’hExo and the individual nucleotides from positions 18–26 observed in the phosphorylated ternary complex (P-3°) and dephosphorylated ternary complex (3°) simulations with the wild-type stem-loop (WT), the uridylated intermediate (5U), the truncated stem-loop (Trunc), and the adenylated stem-loop (5A). Individual frames were counted once per interaction (e.g. a frame with three different interactions between the protein and that nucleotide would have been counted three times). Hydrogen bonds were analysed with VMD based on a donor-acceptor distance of 3.5 Å and an angle cut-off of 30°.

Position	WT (P-3°)	WT (3°)	5U (P-3°)	5U (3°)	Trunc (P-3°)	Trunc (3°)	5A (P-3°)	5A (3°)
18	317	455	337	734	1338	1156	294	76
19	823	898	1073	1277	1045	758	606	494
20	1208	1683	1932	1417	NA	NA	665	1021
21	1011	916	951	1274	NA	NA	667	656
22	176	345	96	1088	NA	NA	998	175
23	520	658	800	631	NA	NA	287	138
24	1317	588	3559	1344	NA	NA	329	247
25	1723	2294	NA	NA	NA	NA	NA	NA
26	2040	909	NA	NA	NA	NA	NA	NA

In the ternary complex with phosphorylated SLBP, 3’hExo has more interactions with the final nucleotide in the uridylated intermediate (a uracil at position 24) than it does with the final nucleotide in the wild-type stem-loop (an adenine at position 26), even though the uridylated intermediate is two nucleotides shorter than the wild-type stem-loop. The adenylated stem-loop has greatly reduced interactions with 3’hExo relative to both the wild-type and uridylated RNAs, showing that this effect is specific to uridylation. Looking further up the stem, to the final two nucleotides shared by all four of our simulation RNA molecules (nucleotides 18–19 in Table 3), we see an increase in interactions for the truncated stem-loop, where these nucleotides are terminal, relative to the other three stem-loops, where they are internal.

In these same simulations, with the phosphorylated SLBP, we also observe that 3’hExo shows increased interactions with the two nucleotides at the bottom of the stem (C20 and 21 in the wild-type stem-loop, U20 and 21 in the uridylated intermediate) relative to the two nucleotides immediately after, which form the first portion of the 3’ flanking sequence. Noticeably, this increase in interaction at the bottom of the stem aligns with the site of truncation/uridylation from the first round of partial degradation, and may help explain exactly how these specific intermediates are being generated during the early stages of histone mRNA degradation.

**Figure 6.**
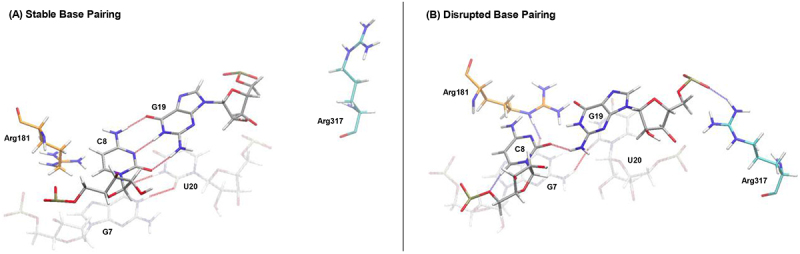
Representative frames from the 5U-SLBP-3’hExo (5U 3°) simulation showing (A) stable and (B) destabilized base pairing between C8 and G19 (grey). The G7-U20 base pair (grey), Arg181 of SLBP (orange), and Arg317 of 3’hExo (cyan) are also depicted. Intermolecular hydrogen bonds are shown in blue, and intramolecular hydrogen bonds are shown in red. All hydrogen bonds were predicted in VMD based on a donor-acceptor distance of 3.5 Å and an angle cut-off of 30°. When the C8-G19 base pair is disrupted, the G7-U20 base pair also tends to be disrupted. C8 shifts away from G19 out of the base pair, favouring base stacking with G7, which interacts with Arg181 when shifted out of position. Arg317 of 3’hExo tends to interact with the backbone of G19 when the C8-G19 base pair is disrupted.

The dephosphorylation of SLBP also has a noticeable effect on these interactions. There is a slight reduction of RNA–protein interactions for each stem-loop intermediate when compared to the simulations with phosphorylated SLBP. The only two nucleotides that exhibit an increased interaction in these simulations are the two nucleotides that immediately precede the truncation/uridylation, A18 and G19. This increase in interactions specifically is only seen in the wild-type and uridylated stem-loops, and it is more pronounced in the uridylated intermediate. The best explanation for this is shown in the data in Table 2, which demonstrates how uridylation weakens the base-pairing in the stem-loop as far up as the C8-G19 base pair, beyond the two G-C base-pairs at the bottom that have been replaced with non-canonical G-U pairs. Figure 6 helps further depict this effect, as the representative frames from our simulation depict the way that both the disruption of the G7-C/U20 base pair (a disruption most commonly seen when the stem-loop is uridylated and SLBP is dephosphorylated) and Arg317 of 3’hExo facilitate the weakening of the C8-G19 base pair. As the base-pairing weakens throughout the stem, the RNA presents more single-stranded targets for 3’hExo to degrade, increasing interactions between the protein and RNA all the way through the stem as shown in Table 3.

Taken as a whole, these experiments provide more insight into how the enzyme may generate these partially degraded and uridylated intermediates and more evidence for the idea that while uridylation may be briefly stabilizing the stem-loop and allowing the ternary mRNA-protein complex to maintain its shape, it also primes the RNA molecule for further degradation by 3’hExo.

## Discussion

In this study, we integrate experimental and computational techniques to investigate how the trimming and uridylation of the histone mRNA stem-loop affects its binding interactions with SLBP in both phosphorylation states of the TPNK motif and 3’hExo. Ultimately, this will help us to better understand the role of both histone mRNA uridylation and the dephosphorylation of SLBP in the TPNK motif during histone mRNA degradation. Our fluorescence polarization assays (Figure 2B) provide evidence that while SLBP is capable of binding the partially degraded and uridylated stem-loops, binding is significantly reduced with the intermediates, while our EMSAs (Figure 2C) indicate that 3’hExo remains bound to the partially degraded and uridylated stem-loops. These observations pave the way for a more detailed analysis of these RNA-protein complexes in our molecular dynamics simulations.

One key finding from these simulations was that the 3’ uridylation of histone mRNA maintains the overall shape of the wild-type stem-loop, as shown by the RMSD analysis of nucleotides 1–19 (Figure 3). This effect is unique to uridylation, as a control simulation run on an adenylated stem-loop appears to take on a slightly different conformation than the wild-type and uridylated stem-loops. Despite this, MINT analysis of base pair occupancy (Table 2) shows that uridylation weakens base pairs in the stem that are not otherwise weakened, especially when the histone mRNA has been dephosphorylated. This may partially explain the reduced binding affinity seen in our fluorescence experiments (Figure 2). Further, these simulations also show that when the TPNK motif of SLBP’s RNA-binding domain is dephosphorylated, the key G7–Arg181 interaction is reduced much more for the uridylated stem-loop than for the wild-type, truncated, or adenylated control. This suggests that histone mRNA uridylation and dephosphorylation of SLBP may be working together to weaken SLBP’s affinity for histone mRNA.

On the other side of the stem-loop, uridylation serves two functions. By extending the mRNA beyond the initial degradation into the stem-loop by 3’hExo, uridylation serves to provide an RNA platform for 3’hExo to continue to interact with. This effect can be seen visually in Figure 5 and is quantified in Table 3. It is also specific to uridylation, as it is not replicated in our adenylation controls. As degradation proceeds and SLBP is dephosphorylated, it appears that 3’hExo also has increased access further up the stem even beyond the site of uridylation/truncation from the initial round of degradation. This aligns with observations that uridylation of the stem-loop and dephosphorylation of SLBP have a destabilizing effect on the stem-loop beyond replacing G-C base-pairs with G-U base-pairs.

Based on these results, we propose a model of histone mRNA degradation in which the uridylation of the stem-loop plays a key role in weakening the stem-loop and its interactions with SLBP while also allowing 3’hExo to remain associated with the mRNA and possibly priming the mRNA for further degradation by 3’hExo. During S phase, the stem-loop at the 3’ end of histone mRNA is bound by SLBP that is phosphorylated at the TPNK motif in its RNA-binding domain [19]. 3’hExo also binds to the stem-loop, forming a ternary complex with the RNA and SLBP [15]. Prior to degradation, trimming by 3’hExo into the stem is impeded by the structure of the stem-loop, which is stabilized by phosphorylated SLBP. Any bases removed by 3’hExo at this point in the histone mRNA lifecycle are quickly replaced by uridylation [9]. Before degradation begins, SLBP is dephosphorylated [25], which weakens its binding affinity for the stem-loop by 7–11-fold [19,23]. This may destabilize the RNA-SLBP complex enough to allow 3’hExo to degrade into the stem, and our fluorescence experiments confirm that this partial degradation would destabilize the RNA-SLBP complex further. This destabilization may be what allows TUT7 access to uridylate the intermediate all the way into the stem [9].

Our simulation results combined with the fluorescence polarization indicate that while this uridylation may be enough to restore some key structural features of the mRNA stem-loop and key interactions between the RNA and SLBP, uridylation of the mRNA ultimately decreases the interaction between the RNA and the protein. This effect is exacerbated by the dephosphorylation of SLBP, which has a stronger effect on the uridylated intermediate than the wild-type stem-loop. This effect may be further magnified by 3’hExo, which has more interactions with the 3’ end of the uridylated intermediate than the nucleotides at the 3’ end of the wild-type stem-loop, even though this stem-loop is two nucleotides shorter than the wild-type. In fact, when SLBP is dephosphorylated and the histone mRNA is uridylated, our simulations show that 3’hExo has increasing access well into the stem, which may help explain how the enzyme further degrades the histone mRNA stem-loop as degradation proceeds.

Altogether, this study provides a more detailed account of the role of the short oligo(U) tails commonly found during degradation compared to previous studies, which postulated that uridylation by TUT7 may serve to either create an oligo(U) tail that acts as a binding site for the Lsm1-7 complex [11] or to stimulate 3’ to 5’ degradation by the exosome [12]. SLBP must dissociate from the stem-loop for histone mRNA degradation to proceed to completion [50], but the presence of these short U-tails found throughout the stem-loop during degradation indicates that this may not be an immediate process. Our results show that uridylation weakens SLBP’s affinity for the stem-loop, especially following TPNK dephosphorylation. At the same time, dephosphorylating SLBP and adding a U-tail to the 3’ end of histone mRNA increases 3’hExo’s ability to interact with the histone mRNA, both at the 3’ end and well into the stem-loop, which may also contribute to the weakening of the base pairing throughout the stem. The fact that our simulation with the truncated version of the stem-loop and our control simulation with an adenylated stem-loop did not display similar results is evidence that this effect is specific to uridylation. Taken together, all of these observations from our simulations provide more context to the model proposed by Meaux et al. [2018, 51]; their results suggest that the progression of histone mRNA degradation requires the interaction between SLBP and the stem-loop to change, either through SLBP modification, the helicase activity of Upf1, or both.

Our computational and experimental results confirm that SLBP dephosphorylation is capable of altering the structure of the histone mRNA stem-loop and its interaction with 3’hExo. They also show that the activity of TUT7 during degradation is responsible for more than just priming for the Lsm1-7 complex. Uridylated intermediates maintain the overall shape of the RNA stem-loop, allow 3’hExo to remain in contact with the 3’ end of the histone mRNA, and weaken SLBP’s affinity for the stem-loop. When uridylation and the dephosphorylation of SLBP are combined, Watson–Crick base-pairs in the stem appear to weaken as well. As prior results have linked uridylation to degradation, both with histone mRNA and poly(A) mRNA [8,9,52], these results indicate that this may be a process that bridges the gap between a fully stable wild-type mRNP and a totally dissociated one that is being actively degraded.

## Supplementary Material

Supplemental Material

## Data Availability

The data that support the findings of this manuscript are available upon reasonable request from the corresponding author, PEL.
