# Supplementary material for: Uridylation of the histone mRNA stem-loop weakens binding interactions with SLBP while maintaining interactions with 3’hExo
Source: RNA Biol. 2023 Jul 30;20(1):469–81. doi: 10.1080/15476286.2023.2171760 (PMC10388802; doi:10.1080/15476286.2023.2171760)
Supplement: Supplemental Material [file KRNB_A_2171760_SM6494.zip › supplementary_files_2171760_1689923385036/2171760_Supplementary.zip!/Supporting information.docx]

**Supporting information**

**Figure S1.** Secondary structures of **(A)** the wild-type stem-loop (WT), **(B)** the uridylated intermediate (5U), **(C)** the truncated stem-loop (Trunc), and **(D)** the adenylated stem-loop (5A).

**Figure S2.** Root mean square deviation (RMSD) for nucleotides 8-19 of the wild-type stem-loop (WT) (gray), the uridylated intermediate (5U) (red), the truncated stem-loop (Trunc) (blue), and the adenylated stem-loop (5A) (purple) in **(A)** RNA-pSLBP-3’hExo (P-3°) and **(B)** RNA-SLBP-3’hExo (3°) simulations. For each trajectory, all heavy atoms of nucleotides 8-19 were aligned to the stem-loop from the 4QOZ reference structure. RMSD was calculated in VMD for all heavy atoms of nucleotides 8-19 based on 1000 frames over each 1000-ns simulation.

**Figure S3. (A)** Root mean square deviation (RMSD) for nucleotides 1-19 of the wild-type stem-loop (WT) (gray), the uridylated intermediate (5U) (red), the truncated stem-loop (Trunc) (blue), and the adenylated stem-loop (5A) (purple) in RNA-SLBP-3’hExo (3°) simulations. All heavy atoms of nucleotides 8-19 were aligned to the 4QOZ reference structure, and RMSD was calculated in VMD for all heavy atoms of nucleotides 1-19 based on 1000 frames over the 1000 ns of simulation time. **(B)** Schematic of the histone mRNA stem-loop with nucleotides used for alignment highlighted in gray (top) and the 4QOZ reference structure used for alignment (bottom). **(C)** Overlay of average structures of WT (gray), 5U (red), Trunc (blue), and 5A (purple).

**Figure S4.** Box plot of base pair data from the RNA-pSLBP-3’hExo and RNA-SLBP-3’hExo simulations. Base pair data were calculated using MINT software (44).

**Table S1.** DNA templates (TriLink Biotechnologies) used for *in vitro* transcription of the wild-type stem-loop (WT) and uridylated intermediate (5U) constructs. For each construct, the CTOP sequence is underlined in red and the template used to transcribe the RNA construct is shown in black.

**Table S2.** Occupancy values for intermolecular hydrogen bonds between the RNA and SLBP in RNA-pSLBP-3’hExo (P-3**°**) and RNA-SLBP-3’hExo (3**°**) simulations with the wild-type stem-loop (WT), the uridylated intermediate (5U), the truncated stem-loop (Trunc), and the adenylated stem-loop (5A). Hydrogen bonds were predicted in VMD based on a donor-acceptor distance of 3.5 Å and an angle cutoff of 30°.

Available at: https://figshare.com/s/1d5ebc885f261895d06b

**Table S3.** Occupancy values for intermolecular hydrogen bonds between the RNA and 3’hExo in RNA-pSLBP-3’hExo (P-3**°**) and RNA-SLBP-3’hExo (3**°**) simulations with the wild-type stem-loop (WT), the uridylated intermediate (5U), the truncated stem-loop (Trunc), and the adenylated stem-loop (5A). Hydrogen bonds were predicted in VMD based on a donor-acceptor distance of 3.5 Å and an angle cutoff of 30°.

Available at: https://figshare.com/s/1d5ebc885f261895d06b

**Table S4.** Grubbs’ outlier test results for base pair data from the RNA-pSLBP-3’hExo and RNA-SLBP-3’hExo simulations. The 61.60% (C8-G19 base pair) value was identified as an outlier based on a significance level of 0.01.
